# Study on a Chiral Structure with Tunable Poisson’s Ratio

**DOI:** 10.3390/ma14123338

**Published:** 2021-06-17

**Authors:** Yanming Fu, Tianbiao Yu, Xin Wang

**Affiliations:** 1 Laboratory Management Center, Shenyang Sport University, Shenyang 110102, China; fym8188@163.com; 2 School of Mechanical Engineering and Automation, Northeastern University, Shenyang 110819, China; tianbiaoyudyx@gmail.com; 3 School of Kinesiology, Shenyang Sport University, Shenyang 110102, China

**Keywords:** negative Poisson’s ratio, TPU, elastic properties, structural

## Abstract

A chiral structure with a negative Poisson’s ratio containing a hollow circle with varying diameters was designed, and the finite element method was used to investigate the variation in the Poisson’s ratio when the hollow circle diameter was varied (*d* = 0, 1, 2, 3, and 4 mm). The simulation results showed that the Poisson’s ratio was sensitive to the hollow circle diameter, and the minimum Poisson’s ratio was −0.43. Three specimens with different hollow circle diameters (*d*′ = 0, 1, and 3 mm) were 3D-printed from thermoplastic polyurethane, and the Poisson’s ratio and equivalent elastic modulus were measured. In the elastic range, the Poisson’s ratio increased and the equivalent elastic modulus decreased as the hollow circle diameter increased. The simulation and experimental results showed good agreement. The proposed structure is expected to be applicable to protective sports gear owing to its high energy absorption and the fact that its properties can be modified as required by adjusting the geometric parameters of the unit cell.

## 1. Introduction

The unusual mechanical properties of mechanical metamaterials are derived from their structural characteristics rather than their composition. Their unique structure confers advantages over many natural or traditional materials, which gives them wide applicability in many fields, such as industry, medicine, sports protection, and daily life [1,2]. Auxetics are metamaterials with a negative Poisson’s ratio and have recently gained much research attention [3]. Auxetics have long been found in nature and include examples such as cubic crystals [4,5], zeolite [6], silicates [7], cat skin [8], and cow teat skin [9]. The study of synthetic isotropic materials with a negative Poisson’s ratio originated in the late 1980s [10]. For isotropic auxetics, the Poisson’s ratio is in the range of −1 to 1 for two-dimensional structures and −1 to 0.5 for three-dimensional structures [1,11,12,13,14]. Auxetics include many classical structures, such as chiral structures, concave structures, missing rib models, and star structures [13,15,16,17,18,19,20,21,22,23,24,25,26,27,28]. Research on auxetics is still in the experimental stage, and there has been little practical application [29]. The effect of a negative Poisson’s ratio on chiral structures and missing rib models is very obvious [3,11,30,31,32,33]. Auxetics can convert vertical forces into horizontal forces through structural changes [34,35], which makes them more resistant to indentation and grants them greater energy absorption than traditional materials.

**Figure 1:**
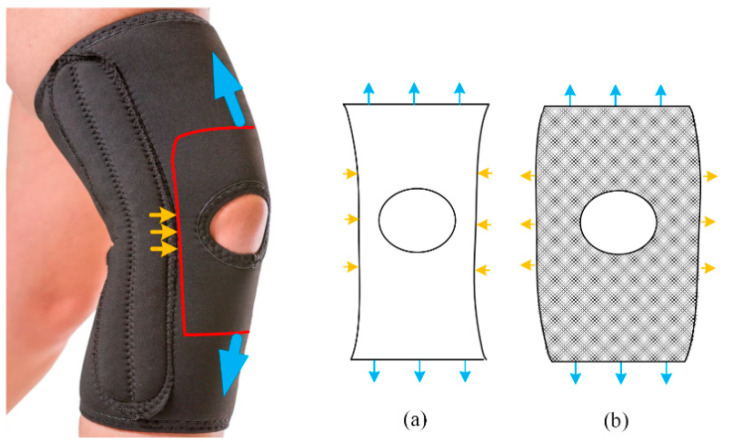
The negative Poisson’s ratio effect of protective gear. (**a**) Tensile effect of ordinary materials. (**b**) Tensile effect of negative Poisson’s ratio material.

The design of protective gear for sports must satisfy strict requirements for comfort and protection [36]. Applying an auxetic to the design of protective gear may improve the cushioning effect, adhesion, and comfort. As an example, the upper and lower ends of a knee protector stretch when the knee flexes. Figure 1a shows the effect of stretching on an ordinary structure. By contrast, Figure 1b shows that the auxetic has good adhesion on both sides of the patella. The bilateral edge effect allows the auxetic to adhere to the knee joint without affecting the position and flexibility of the brace. Of course, this is only a superficial example of the design of a knee protector. It is not a protective gear that we want to design.

In this study, a new auxetic that combines the chiral structure with the missing rib model to obtain approximately symmetric deformation was developed. This may be applicable to the design of protective gear [36,37]. Unlike other chiral structures and missing rib models, the proposed structure intentionally includes hollow circles for central reinforcement and *h* = *D*/2. Such a unique structure may improve symmetry and obtain a suitable Poisson’s ratio. In this study, the finite element method (FEM) was used to calculate the Poisson’s ratio and elastic modulus of the proposed structure and to evaluate the effect of the geometric parameters on the Poisson’s ratio. Then, corresponding specimens were 3D-printed, and a tensile test was performed to measure the mechanical properties and compare them with the simulation results.

## 2. Simulation

**Figure 2:**
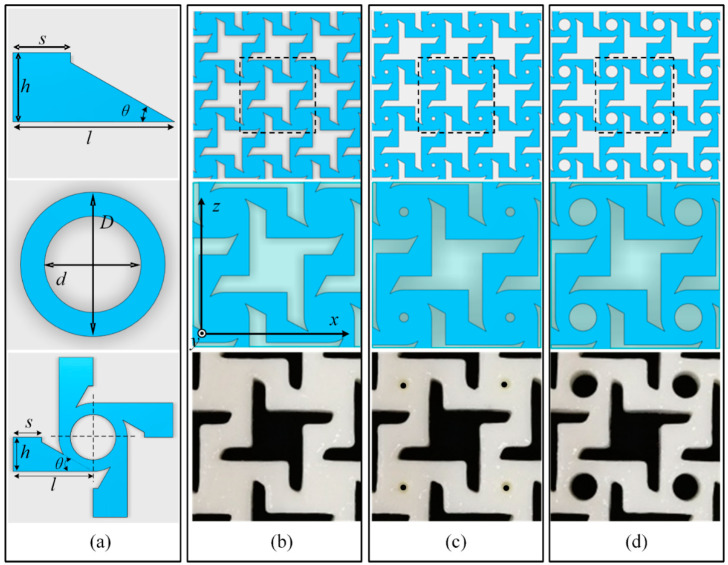
Dimensions and printing model of the unit cell: (**a**) basic shapes and sizes; (**b**–**d**) assembled and printed models with hollow circle diameters of *d* = (**b**) 0, (**c**) 1, and (**d**) 3 mm.

**Figure 3:**
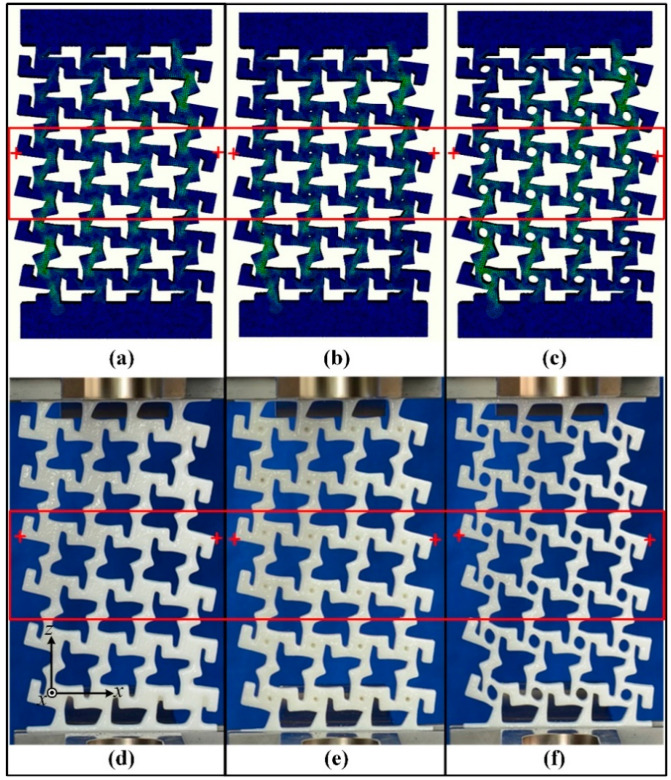
Models in Abaqus with hollow circle diameters of *d*′ = (**a**) 0, (**b**) 1, and (**c**) 3 mm; 3D-printed specimens for tensile tests with hollow circle diameters of *d* = (**d**) 0, (**e**) 1, and (**f**) 3 mm.

As shown in Figure 2, the unit cell of the proposed structure is similar to that of a windmill, and the central position has a circular hole of varying diameters (*d* = 0, 1, 2, 3, and 4 mm). The rest of the unit cell parameters were set to *s* = 2.5 mm, *l* = 7 mm, *h* = 3 mm, and *θ* = arctan 4/7. *D* is the diameter of the circle, and d is the diameter of the hollow circle. The length was 6 mm, and the thickness of the model was 5 mm. The cellular structure and assembly of the model were drawn in the software Autodesk 123D (Autodesk, Inc., San Rafael, CA, USA). The FEM analysis of the structure with varying hollow circle diameters (*d* = 0, 1, 2, 3, and 4 mm) was per-formed in Abaqus. In the simulation, the model was 90 mm long (six repeating units and 7 mm edge margins at the top and bottom), 49.5 mm wide (four repeating units), and 5 mm thick. Figure 3 shows the meshes of three models generated with Abaqus: (a) 657,067 units (C3DR8, 748,674 nodes), (b) 645,490 units (C3DR8, 738,144 nodes), and (c) 615,791 units (C3DR8, 711,450 nodes). All meshes had a size of 0.3. The bottom of the model was completely fixed by a clamp. The vertical displacement of the top of the model was set to 1 mm/step. The left and right sides of the model were allowed to move freely. The simulation only considered linear deformation; nonlinear and large deformations were not considered.

Some parameters were varied to evaluate the influence on the Poisson’s ratio. Because the objective was to realize a potential application to protective gear, the structure needed to be symmetric, so *h* = *D*/2 could not be changed. *θ*, *s*, and *l* are related to each other; when s is fixed, the range of *θ* is limited and *s* changes with *l*. Thus, only *l* and *θ* were varied to evaluate the effect on the Poisson’s ratio.

## 3. Experiment

Three types of specimens were fabricated from thermoplastic urethane (TPU, Lubrizol, Wickliffe, OH, USA) (density of 1.2 g/cm^3^, elastic modulus of 30 MPa, and Poisson’s ratio of 0.45) [24] with different hollow circles (*d*′ = 0, 1, and 3 mm) using the 3D printer EP-3850 ((E-plus-3D, Beijing, China). The specimens are shown in Figure 3d–f. The specimens were printed at the highest precision mode available (layer thickness of 0.3 mm and air supply of compressed air). Because of the limited accuracy of the printer, many details were not clearly displayed by the specimens. For example, Figure 3b,e show a model and specimen, respectively, with equivalent parameters (i.e., *d* = *d*′ = 1 mm), but the specimen in Figure 3e has no hollow circle, just a pit. Tensile tests were performed on the specimens (five for each type) using a universal testing machine (Zwick010, Zwick/Roell, Ulm, Germany), where the load was applied along the z direction. As shown in Figure 3d–f, each specimen was clamped on both sides, the bottom was fixed, and the top was moved upward at a speed of 3 mm/min. The left and right sides of the specimen were allowed to move freely. During the stretching process, a camera was used to take pictures of the front side at preset intervals. These images were used to calculate the transverse displacement in the x direction and longitudinal displacement in the z direction during stretching. The results were calibrated by calculating the pixel differences between reference points in the images (red crosses (+) in the red boxes of Figure 3).

## 4. Results and Discussion

**Figure 4:**
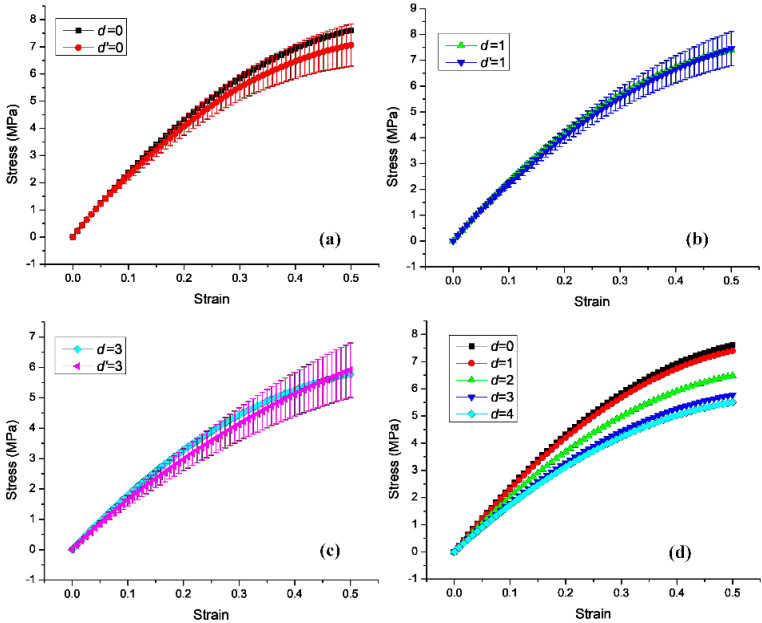
Stress–strain curves of the Abaqus simulation and tensile tests, where d represents the simulation results and d′ represents the experimental results: (**a**) *d* = *d*′ = 0, (**b**) *d* = *d*′ = 1 mm, and (**c**) *d* = *d*′ = 3 mm. (**d**) Stress–strain curves of the five structures (*d* = 0, 1, 2, 3, and 4 mm) simulated in Abaqus.

The simulation results in Abaqus were used to obtain the stress–strain curve in the y direction. The model was in the elastic range when the displacement was less than 15 mm (strain was less than 0.2). Figure 4d shows the stress–strain curves obtained in the simulation (*d* = 0, 1, 2, 3, and 4 mm). Figure 4a–c shows the corresponding stress–strain curves in the tensile tests (*d*′ = 0, 1, and 3 mm).

### 4.1. Simulation Results

**Figure 5:**
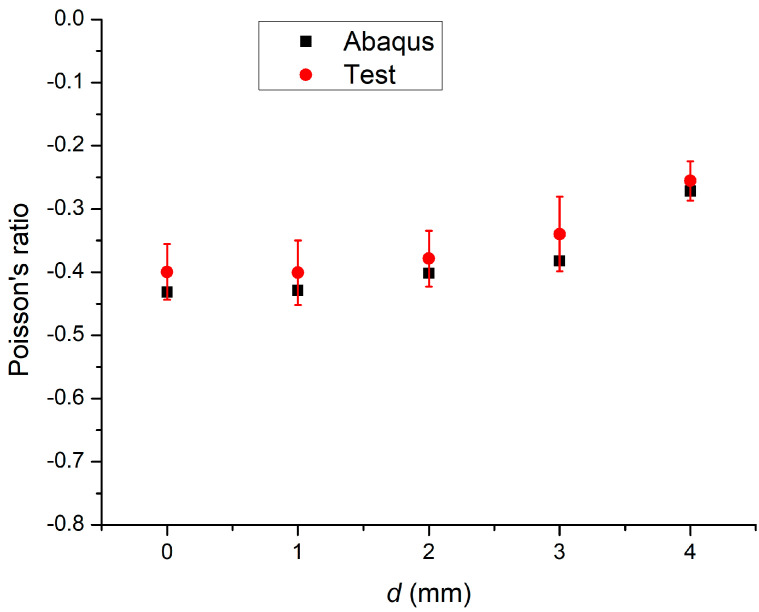
Poisson’s ratio in the Abaqus simulation and tensile tests.

The Poisson’s ratio, *ν*, was defined as the ratio of the transverse contraction strain to the longitudinal extension strain in tension (i.e., *ν* = −ε_x_/ε_y_, where ε_x_ and ε_y_ are the transverse contraction strain and longitudinal extension strain, respectively). According to the strain data along the x- and *z*-axis directions, the Poisson’s ratios at *d* = 0, 1, 2, 3, and 4 mm were −0.43, −0.42, −0.40, −0.38, and −0.27, respectively (Figure 5). At the same displacement, the Poisson’s ratio increased up to 37.2% with increasing *d* and the tension decreased. The elastic modulus decreased with increasing *d* (i.e., 22.64, 22.01, 19.30, 17.18, and 16.39 MPa, respectively). The elastic modulus was obtained from the slope of the stress–strain curve at strains of 0.05 and 0.1.

**Figure 6:**
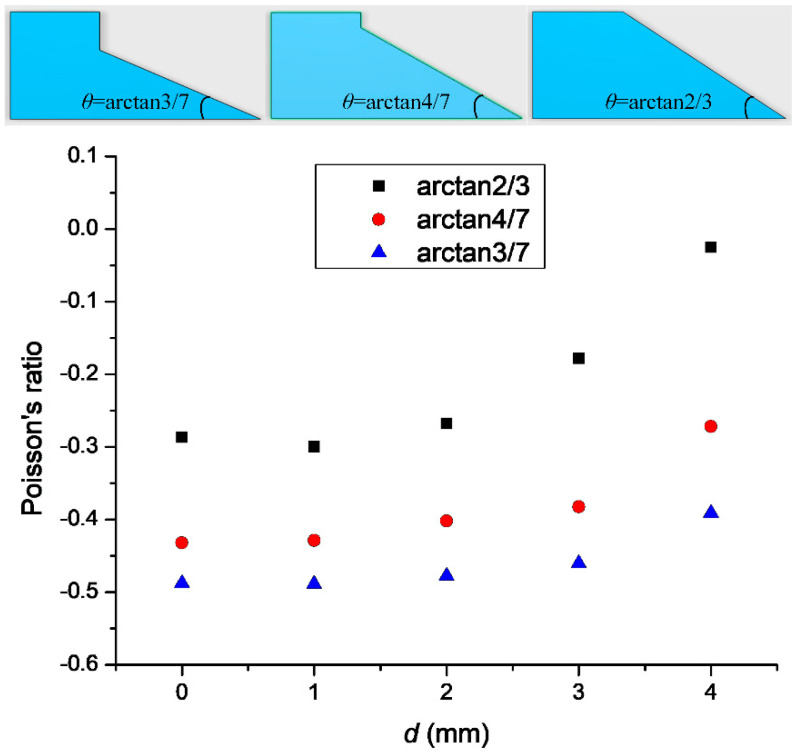
The relationship between *θ* and Poisson’s ratio.

Models with different *θ* and different mesh sizes were also considered in the simulation. As shown in Figure 6, *θ* = arctan 4/7, *θ* = arctan 2/3, and *θ* = arctan 3/7 were considered. The Poisson’s ratio increased 38.7–93.6% with increasing *θ*. Each curve showed the same trend. Increasing *θ* reduced the rotation effect of the cell structure, which explains the increase in the Poisson’s ratio.

**Figure 7:**
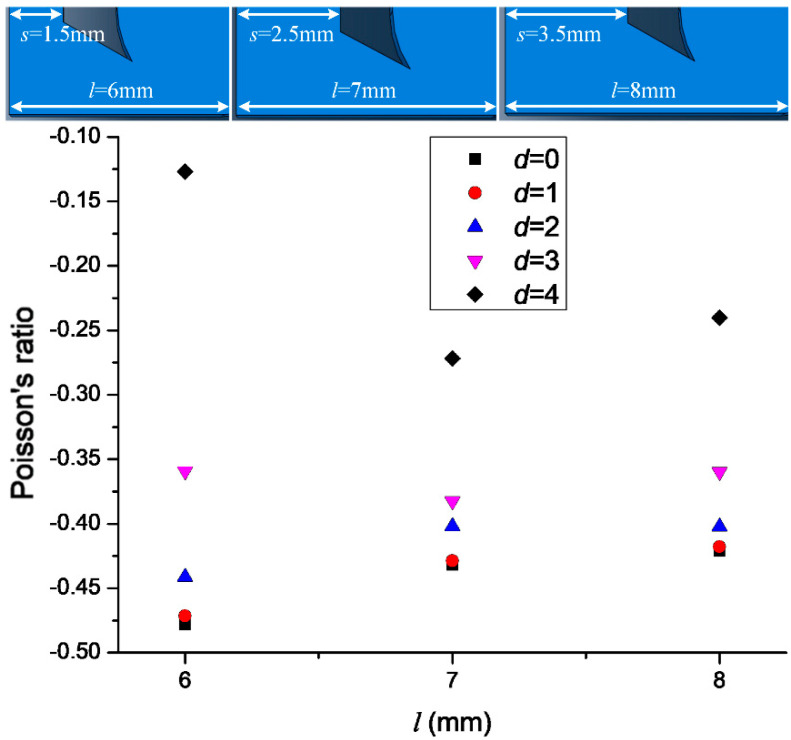
Relationship between l and Poisson’s ratio.

Changing *l* was also found to affect the Poisson’s ratio. As shown in Figure 7, *l* = 7, 6, and 8 mm were considered. Although the Poisson’s ratio increased with *d* for all three *l* values, the range of the increase was different. The *l* = 6 mm model had the greatest range for the Poisson’s ratio (−0.1 to −0.5), followed by the *l* = 8 mm model (−0.2 to −0.4) and then the *l* = 7 mm model (−0.3 to −0.4). Thus, varying *l* or *s* affected the Poisson’s ratio of different models, and the trends were consistent. The most suitable Poisson’s ratio can be obtained by adjusting *l* or *s*. According to the design requirements of protective gear, the structure with the smallest variation in the Poisson’s ratio is the ideal choice. However, the simulation results still needed to be verified against the experimental results.

**Figure 8:**
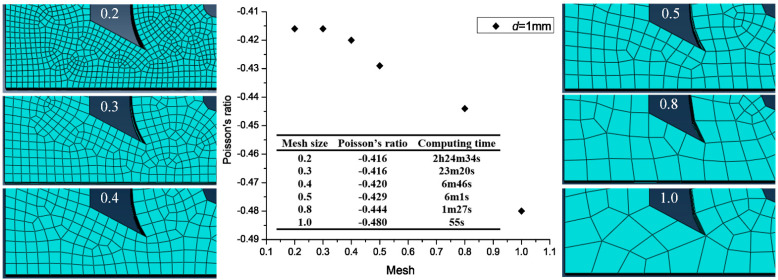
Relationship between the mesh size and Poisson’s ratio using the model with the hollow circle diameter *d* = 1 as an example.

The Poisson’s ratio was affected by the mesh size. Different mesh sizes were considered: 0.2, 0.3, 0.4, 0.5, 0.8, and 1. As an example, Figure 8 shows the relationship between the mesh size and Poisson’s ratio at *d* = 1 mm. Although the Poisson’s ratio decreased with increasing mesh size, the Poisson’s ratio remained the same at mesh sizes of 0.3 and 0.2 (−0.416). This indicates that a mesh size of 0.3 was conducive for calculation accuracy.

### 4.2. Experimental Results

Figure 5 shows that, in the elastic tensile range, the Poisson’s ratios at *d*′ = 0, 1, 2, 3, and 4 mm had mean values and standard deviations of −0.40 ± 0.04, −0.40 ± 0.05, −0.37 ± 0.04, −0.23 ± 0.05, and −0.25 ± 0.03, respectively. Figure 4a–c compares the stress–strain curves of three structures obtained in the tensile tests and simulations. The results showed that the simulated Poisson’s ratios were within the range of values obtained from five tensile tests of a specimen with the same structure. Thus, the simulation and experimental stress–strain curves showed good agreement. In other words, the Poisson’s ratios of the tensile test specimens in the elastic range were similar to those in the simulation results.

### 4.3. Discussion

The simulation and experimental results showed that the proposed auxetic made of TPU offers good energy absorption, which may help protective gear stabilize a joint. A joint protector made from an auxetic may be more flexible and comfortable than one made from traditional material. Different mechanical properties can be obtained by modifying the unit cell parameters as required. The proposed auxetic can be applied in assembling and designing products with a wide range of applicability in sports equipment, sports facilities, and protective gear [36,37,38].

## 5. Conclusions

An auxetic, where the unit cell contained a hollow circle with varying diameter, was proposed, and the properties were evaluated using FEM simulations and tensile tests. The simulation results showed that the Poisson’s ratio could be adjusted in the range of −0.43 to −0.27, and the experimental results showed good agreement with the simulation results. Adjusting *θ* and *l* subtly altered the Poisson’s ratio. Reducing *θ* decreased the Poisson’s ratio, but a smaller *θ* also greatly reduced the tensile strength of the structure. Reducing *l* also decreased the Poisson’s ratio, but the range of Poisson’s ratio increased for *d* = 0–5 mm. Therefore, *θ* and *l* should be adjusted according to the required tensile strength. Although the mesh size was found to have some influence on the Poisson’s ratio, a mesh size between 0.2 and 0.4 offered sufficient calculation accuracy. Further increasing the calculation accuracy would also increase the calculation time. The proposed auxetic is expected to be applicable to the design of joint protectors with varying structures and mechanical properties.

## Data Availability

Not applicable.
